# Preserving residual kidney function in peritoneal dialysis: from conventional approaches to contemporary practice

**DOI:** 10.1093/ckj/sfag131

**Published:** 2026-04-25

**Authors:** Haci Hasan Yeter

**Affiliations:** Department of Nephrology, Hacettepe University Faculty of Medicine, Sihhiye, Ankara, TÜRKİYE

**Keywords:** artificial intelligence, glucose exposure, incremental peritoneal dialysis, kidney function, peritoneal dialysis, remote monitoring, residual

## Abstract

Residual kidney function (RKF) is a key determinant of outcomes in patients undergoing peritoneal dialysis (PD), contributing to solute clearance, volume regulation, reduced inflammation, and improved survival. Nevertheless, RKF commonly declines after PD initiation, often due to potentially modifiable clinical and treatment-related factors. This narrative review summarizes current evidence on the mechanisms underlying RKF loss in PD and evaluates established and emerging strategies to preserve RKF in contemporary practice. Major contributors to RKF decline include hemodynamic instability, cumulative glucose exposure, inflammation and peritonitis, nephrotoxic medications, dialysis-related volume shifts, and kidney disease-specific conditions. Conventional strategies such as incremental and individualized PD prescriptions, use of biocompatible and glucose-sparing solutions, careful volume and blood pressure management, renin–angiotensin–aldosterone system blockade, infection prevention, and tailored management after kidney allograft failure are reviewed. Importantly, current guideline-based strategies largely address these factors in isolation. We propose that RKF preservation should be approached through an integrated, mechanism-based framework, in which cumulative glucose exposure represents a central modifiable mediator linking multiple pathways of kidney injury. In this context, remote monitoring and artificial intelligence-based tools may serve as enabling platforms that integrate longitudinal clinical and treatment data, supporting individualized and proactive care. Preserving RKF should remain a central therapeutic goal in PD, and combining established physiological principles with modern digital technologies may provide a more effective framework for improving long-term patient outcomes.

## INTRODUCTION

Preservation of residual kidney function (RKF) is widely recognized as one of the strongest predictors of favorable outcomes in peritoneal dialysis (PD) [1]. Even small amounts of RKF contribute substantially to solute clearance, volume control, patient well-being, and even survival. Despite this, RKF loss remains common after PD initiation and may be accelerated by potentially modifiable treatment-related factors. Preservation of RKF is also emphasized in contemporary guideline documents, including the updated International Society for Peritoneal Dialysis (ISPD) recommendations for PD prescribing, which recognize it as a key determinant of clinical outcomes and an important target of individualized dialysis care [2].

The landscape of PD has evolved significantly over the past decade. Automated peritoneal dialysis (APD) has become the dominant modality in many regions, while incremental peritoneal dialysis (IPD) has re-emerged as a patient-centered strategy for dialysis initiation [3]. At the same time, glucose exposure has been increasingly implicated in peritoneal membrane injury, systemic inflammation, and possibly accelerated RKF decline [4]. On the other hand, remote patient management systems and artificial intelligence (AI) have the potential to be game changers. These developments emphasize the need to reassess current approaches to preserving RKF in modern PD practice (Table 1).

**Table 1:** Overview of key studies on RKF preservation in PD.

Year	Study	Population/design	Method/outcome	Key findings
2000	Singhal *et al*. [5]	CAPD; cohort	RKF decline	RKF loss associated with male gender, increased BMI, DM, CHF, proteinuria, peritonitis
2001	CANUSA Study [8]	PD; cohort reanalysis	RKF and survival	RKF stronger predictor than peritoneal clearance and 250-ml increment in urine volume associated with 36% decrease in death
2003	NECOSAD-2 [7]	Incident PD; cohort	RKF and outcomes	RKF is strong associated with survival and QoL
2003	Johnson *et al*. [15]	Incident PD; cohort	RKF decline	RKF loss associated with low baseline RKF, increased BMI, high protein intake, and DM
2011	Michels *et al*. [23]	PD; cohort	APD vs CAPD	Faster RKF decline with APD
2012	Guest *et al*. [21]	PD; modeling	IPD	RKF contributes to clearance and associated with lower need for dialysis
2015	Pérez Fontán *et al*. [24]	Multicenter cohort	APD vs CAPD	Higher risk of anuria with APD
2016	Chang *et al*. [31]	RCT	Icodextrin	Better RKF preservation with icodextrin
2016	TRIO trial [29]	RCT	Biocompatible solution	Slower RKF decline
2017	*bal*ANZ trial [25]	RCT/analysis	UF and solutions	High UF → worse RKF; biocompatible beneficial
2018	Htay *et al*. [19]	Systematic review	Biocompatible fluids	Improved urine volume and RKF
2020	Yeter *et al*. [41]	Observational	RM	Improved volume/hemodynamics
2024	Yeter *et al*. [22]	Observational	IPD	Better RKF preservation

BMI: body mass index; DM: diabetes mellitus; CHF: congestive heart failure; QoL: quality of life; RCT: randomized clinical trial; BP: blood pressure.

This review aims not only to summarize evidence but also to propose an integrated, clinically actionable framework for RKF preservation. Importantly, while several strategies discussed in this review are supported by clinical evidence, others, particularly the conceptualization of cumulative glucose exposure as a central mediator and the integration of remote monitoring (RM) and AI, should be considered hypothesis-generating and future-oriented. These concepts aim to provide a framework for future research and clinical innovation rather than definitive practice recommendations.

Although this is a narrative review, a structured literature search was conducted across the PubMed/MEDLINE and Embase databases, covering studies published up to January 2026. Keywords included “peritoneal dialysis,” “residual kidney function,” “glucose exposure,” “incremental dialysis,” and “remote monitoring.” Relevant clinical trials, observational studies, meta-analyses, and guideline documents were prioritized. Reference lists of selected articles were also screened to identify additional relevant studies. The final selection was based on clinical relevance and methodological quality.

### Definition and clinical relevance of RKF

RKF is defined and measured heterogeneously across clinical studies, with important implications for the interpretation of outcomes. The ADEquacy of Peritoneal Dialysis in MEXico study classified patients as anephric when estimated glomerular filtration rate (eGFR) fell below 1 ml/min/1.73 m^2^, whereas other investigations have used thresholds based on daily urine output (<100 ml/day) or creatinine clearance (<1.0 ml/min) [5, 6]. Consistent with contemporary clinical practice guidelines, even minimal residual diuresis exceeding 100 ml/day or an eGFR of at least 1 ml/min/1.73 m^2^ is considered clinically meaningful and warrants preservation, highlighting that RKF should be viewed as a continuous rather than dichotomous variable.

Broadly, RKF can be assessed using three main approaches: urine volume as a surrogate marker of residual diuresis, renal solute clearance (typically urea and creatinine clearance) as a measure of excretory function, and measured GFR or eGFR as an indicator of filtration capacity. However, these approaches capture different physiological aspects of kidney function and are not directly interchangeable.

Consequently, comparisons across studies should be interpreted with caution, as interventions associated with preservation of urine volume may not necessarily demonstrate the same effect on solute clearance or measured GFR. This heterogeneity represents a key limitation in the current literature and emphasizes the need for more standardized definitions in future research.

RKF contributes to multiple domains relevant to PD outcomes, including clearance of uremic solutes beyond small molecules, phosphate and sodium removal, maintenance of euvolemia, reduced inflammatory burden, and improved nutritional status and quality of life [1, 6]. Clinical evidence consistently demonstrates that RKF is a key prognostic determinant in patients receiving both PD and hemodialysis, associated with lower morbidity and mortality. This association has been substantiated by several landmark studies in end-stage renal disease, including the Canada–United States PD Study Group trial, the Netherlands Cooperative Study on the Adequacy of Dialysis, and large cohort analyses [7–9]. Notably, the observed survival advantage was primarily linked to preserved native renal clearance rather than to dialysis-related solute removal [6].

### Determinants of RKF loss

**Figure 1:**
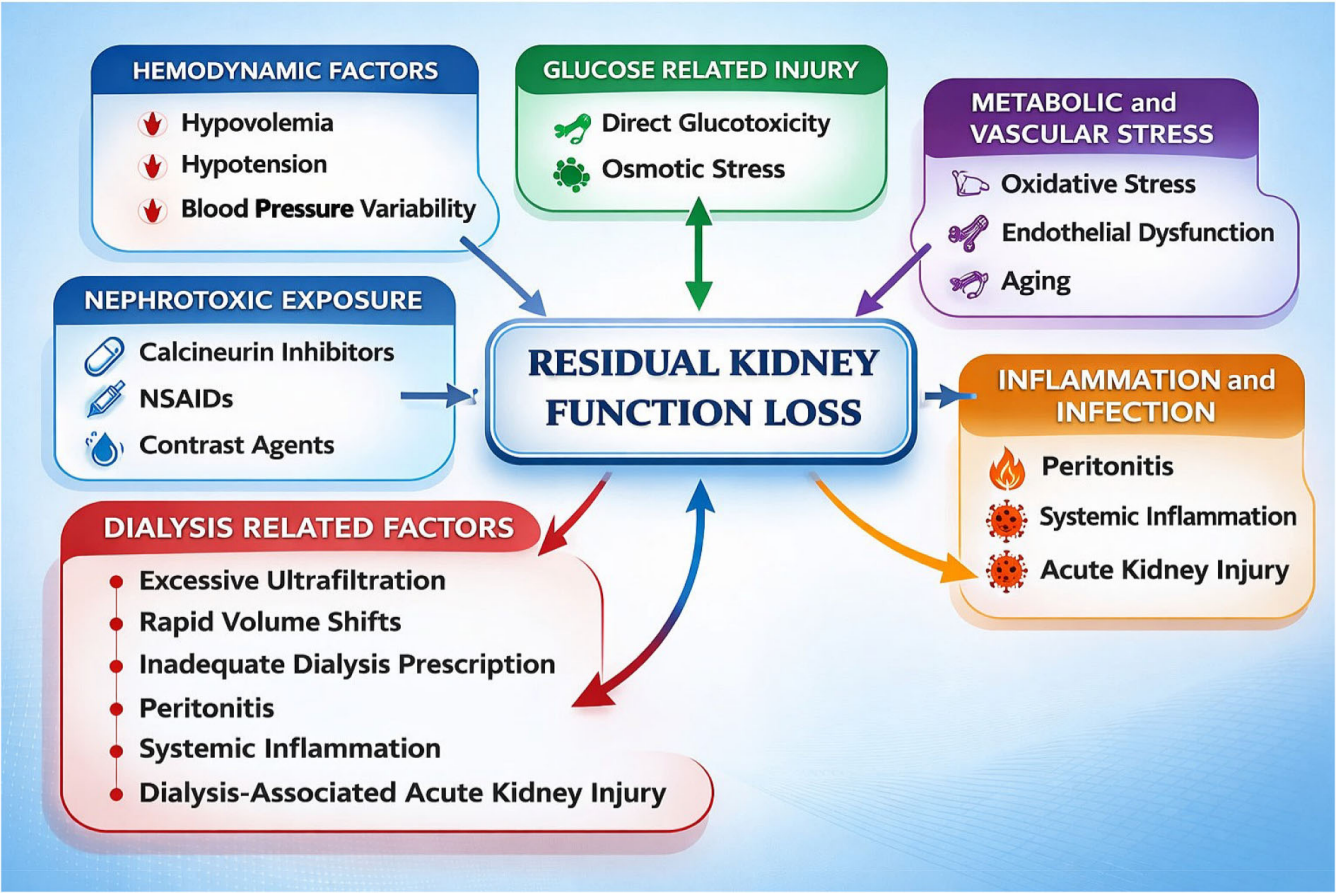
Determinants of RKF loss in PD.

The decline of RKF in PD is not attributable to a single factor but rather reflects the cumulative impact of hemodynamic instability, inflammation, metabolic stress, nephrotoxic drug exposure, and dialysis-related factors that progressively compromise renal perfusion and nephron integrity (Fig. 1).

#### Hemodynamic factors

Hemodynamic disturbances play a central role in RKF loss. In line with Bricker’s intact nephron hypothesis, a reduction in the number of functioning nephrons triggers adaptive responses in the remaining nephrons, including glomerular hyperfiltration and enhanced tubular secretory activity [10]. Excessive dialysis dosing may attenuate these compensatory mechanisms by eliminating the physiological stimuli that drive residual nephron hyperfunction [11]. Also, excessive ultrafiltration (UF), particularly when driven by high-glucose dialysate prescriptions, could lead to repeated episodes of intravascular volume depletion. These subclinical hypovolemic events reduce renal blood flow and predispose residual nephrons to ischemic injury. Rapid fluid shifts further exacerbate renal hypoperfusion in patients with limited autoregulatory reserve.

#### Inflammation and peritonitis

Inflammation represents another key mechanism. Episodes of peritonitis trigger systemic inflammatory responses characterized by cytokine release, oxidative stress, and endothelial dysfunction, all of which negatively affect renal microcirculation [12, 13]. The frequency of peritonitis episodes in PD patients has been identified as an independent risk factor for the development of anuria, with each episode estimated to increase the risk by ~3.8% [6, 14]. This association is biologically plausible and likely multifactorial. Acute peritonitis is frequently accompanied by systemic inflammation, vasodilatation, hypotension, and relative hypovolemia, all of which may compromise renal perfusion and precipitate ischemic injury to residual nephrons [15]. In addition, inflammatory cytokine release during peritonitis may exert direct nephrotoxic effects, further accelerating the decline of RKF. Beyond hemodynamic instability, peritonitis episodes often necessitate temporary modifications in PD prescription, including increased glucose exposure to maintain UF, which may indirectly contribute to RKF loss.

Accumulating evidence suggests that, even in the absence of overt infection, chronic lotw-grade inflammation related to uremia, glucose exposure, and bioincompatible dialysis solutions may contribute to interstitial fibrosis and progressive loss of RKF [16].

#### Cumulative glucose exposure

Repeated and cumulative exposure of the peritoneal membrane to glucose-based dialysis solutions is one of the key mediators of peritoneal membrane injury and decline in RKF through multiple related pathways, including osmotic/metabolic stress, formation of glucose degradation products (GDPs) and advanced glycation end products, oxidative stress and inflammation, angiogenesis, and profibrotic signaling (notably Transforming Growth Factor - beta (TGF-β)) [17]. Over time, these processes may lead to mesothelial cell injury, epithelial/mesothelial-to-mesenchymal transition, extracellular matrix accumulation, microvascular remodeling, and progressive UF failure [18]. High glucose and hypertonic exchanges may further destabilize renal perfusion through repeated osmotic and volume shifts. Clinical studies, including CANUSA and NECOSAD, have demonstrated a dose-dependent association between dialysate glucose exposure and faster RKF loss, whereas glucose-sparing or low-GDP, biocompatible solutions are linked to better preservation of urine output and renal clearance [19]. Thus, peritoneal membrane integrity and RKF preservation appear to be interconnected therapeutic priorities, both influenced by glucose burden.

Beyond its biological effects, cumulative dialysate glucose exposure may be conceptualized as a quantifiable and potentially clinically actionable parameter rather than a passive characteristic of PD prescription. We suggest that glucose exposure be systematically expressed as total grams per day and grams per month, along with the frequency of hypertonic exchanges (e.g. ≥2.27% or ≥3.86% glucose) over time. Additional descriptors, such as the proportion of hypertonic exchanges or average daily glucose concentration, may further refine this assessment. These measures may provide a more physiologically meaningful representation of metabolic and osmotic burden than conventional descriptors, such as modality or the number of exchanges alone.

From a practical standpoint, cumulative glucose exposure may be quantified primarily as prescribed (dwell volume) glucose load, calculated directly from dialysate concentration and exchange volume and expressed as grams per day and grams per month. This approach is simple, reproducible, and readily applicable in routine clinical practice, allowing longitudinal tracking and facilitating prescription adjustments. In research settings, both prescribed and absorbed glucose exposure may be considered. While direct prescribed glucose represents the most standardized and comparable metric across patients and studies, absorbed glucose may better reflect the true metabolic burden. Absorbed glucose can be estimated as the difference between the instilled glucose and the glucose recovered in the effluent. In addition, absorbed glucose can be approximated using commonly available PD adequacy and RM platforms, such as Adequest and ShareSource, which integrate prescription data with peritoneal transport characteristics (e.g. Peritoneal Equilibrium Test (PET) -derived parameters), body surface area, dwell time, and treatment modality to generate estimates of glucose absorption. However, these estimates are model-based and may vary according to underlying assumptions and patient-specific factors.

Accordingly, prescribed glucose should be considered the primary exposure metric, whereas absorbed glucose may serve as a complementary, exploratory parameter in selected research settings. A structured overview of proposed metrics for cumulative glucose exposure in both clinical practice and research settings is provided in Table 2.

**Table 2:** Proposed metrics for cumulative glucose exposure in PD.

	Metric	Definition/calculation	Practical use	Feasibility
Clinical practice or research	Daily glucose exposure (g/day)	[glucose (%) × volume (ml)/100] × dwell count	Routine assessment; prescription adjustment	High
	Monthly glucose exposure (g/month)	Daily glucose × 30	Longitudinal exposure tracking	High
	Hypertonic exposure days	Days with ≥1 exchange ≥2.27% or ≥3.86%	Identify high-intensity prescriptions	High
	Proportion of hypertonic exchanges (%)	Hypertonic exchanges/total exchanges	Treatment intensity indicator	High
Extended (research/exploratory)	Estimated absorbed glucose (g/day)	Instilled glucose − effluent glucose (measured) or estimated based on dwell time and transport	Approximation of true metabolic burden	Moderate

Importantly, interpretation of glucose exposure must account for confounding by indication, as patients requiring higher glucose concentrations are often those with greater comorbidity burden, fluid overload, or UF failure. Therefore, observed associations between glucose exposure and RKF decline may partly reflect underlying clinical complexity rather than a direct causal effect of glucose alone, and should ideally be interpreted in the context of volume status, baseline RKF, and treatment intensity.

**Figure 2:**
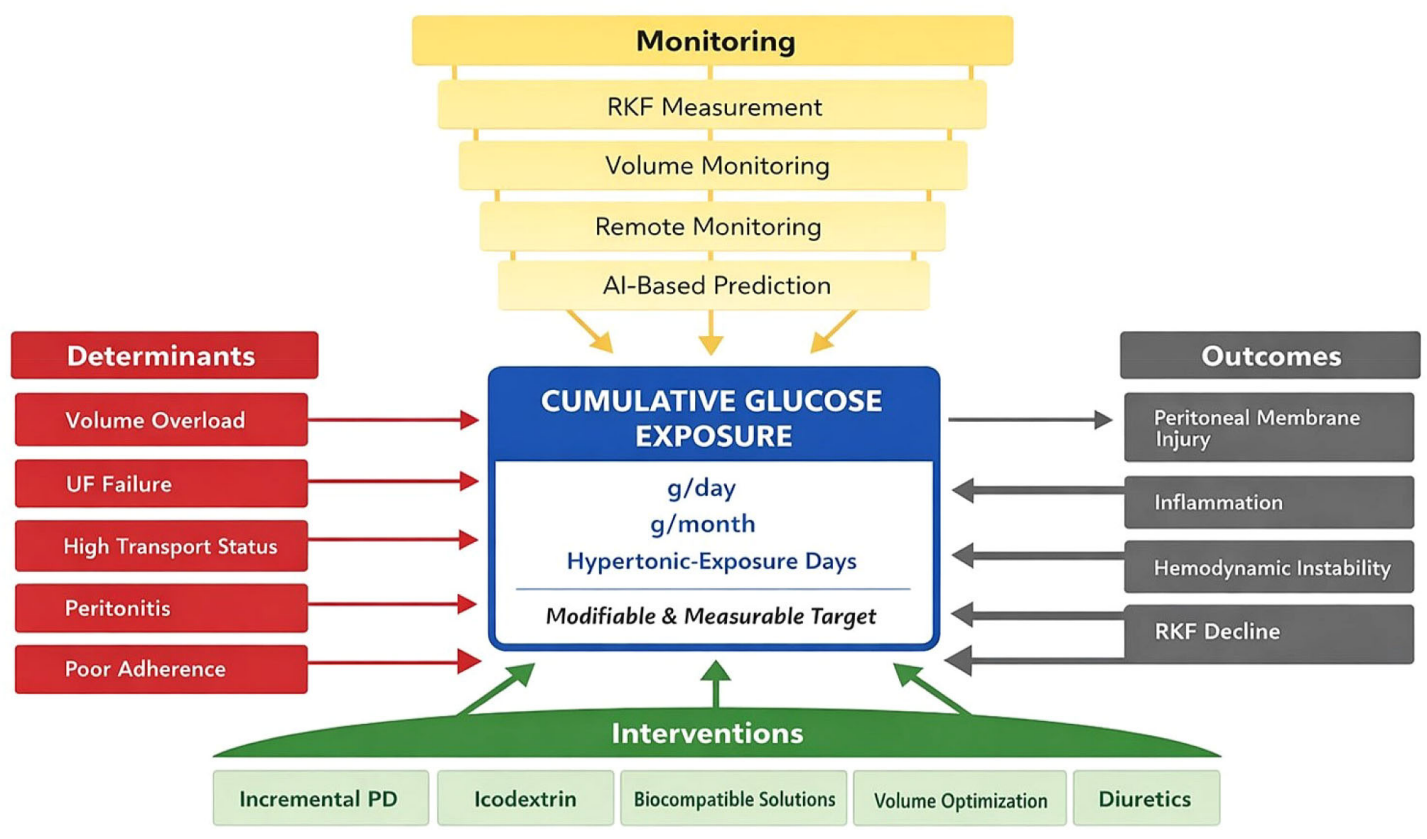
Integrated framework for preserving RKF in PD.

Despite these limitations, integrating cumulative glucose exposure into routine assessment frameworks may help bridge the gap between mechanistic understanding and clinical decision-making. In particular, incorporation of these metrics into longitudinal monitoring strategies and remote patient management platforms may facilitate earlier identification of high-risk exposure patterns and support more individualized, RKF-preserving dialysis prescriptions (Fig. 2).

However, it is important to emphasize that the role of cumulative glucose exposure as a central modifiable mediator of RKF decline remains a conceptual framework supported by mechanistic and observational data, and requires validation in prospective and interventional studies before being adopted as a clinical standard.

#### Nephrotoxic exposures

Nephrotoxic exposures represent an underrecognized but potentially modifiable contributor to RKF decline in patients undergoing PD [1, 6, 12]. Commonly used agents such as nonsteroidal anti-inflammatory drugs, iodinated contrast media, and certain antiviral or chemotherapeutic agents may induce direct tubular injury or impair renal perfusion. Importantly, PD patients are often exposed to these agents during intercurrent illnesses or hospitalizations, where the risk of acute kidney injury is amplified. Even transient episodes of kidney injury may accelerate irreversible loss of residual nephron function. Therefore, systematic avoidance of unnecessary nephrotoxic exposures, along with proactive risk assessment before contrast administration or high-risk pharmacotherapy, should be considered a key component of RKF preservation strategies.

#### Disease-related factors

Disease-related factors remain key determinants of RKF decline after initiation of PD. The trajectory of the underlying kidney disease prior to dialysis initiation is particularly important, as patients with rapidly progressive disease may continue to lose RKF largely independent of dialysis-related factors [7, 8, 15].

Conditions such as diabetic kidney disease, glomerulonephritis, and persistent proteinuric states may drive ongoing nephron loss through sustained inflammation and disease-specific mechanisms [16]. Accordingly, strategies to preserve RKF should extend beyond dialysis prescription and include optimal management of the underlying disease, such as antiproteinuric therapy, glycemic control, and immunosuppressive treatment when appropriate.

These considerations highlight that RKF decline in PD reflects both disease-related and treatment-related processes, and should be interpreted within the context of individual disease trajectories.

### Evidence-based interventions to preserve RKF

Preserving RKF is a key goal in the management of patients undergoing PD, as it is strongly associated with better clinical outcomes.

#### Incremental peritoneal dialysis

IPD is designed to individualize dialysis intensity according to patient-specific requirements, with the dual aims of reducing treatment burden and preserving both peritoneal membrane integrity and RKF [2, 20]. Observational studies and *post hoc* analyses have suggested that less intensive PD prescriptions, particularly during the early stages of therapy, are associated with a slower decline in RKF, likely through improved hemodynamic stability and reduced exposure to glucose-based dialysis solutions [6, 11, 20–22]. However, the evidence supporting a causal relationship between IPD and preservation of RKF remains limited and is largely based on observational data.

Despite these potential benefits, the operational definition of IPD remains highly inconsistent across the literature, substantially limiting interpretation of clinical outcomes and constraining the development of standardized practice recommendations. Although the underlying concept is to adapt dialysis prescription to RKF and metabolic demand, published studies have applied the term using disparate criteria, including reductions in exchange frequency, adjustments in dialysate fill volumes, or flexible treatment schedules based on the proportional contribution of RKF to total solute clearance. While these approaches share the goal of personalizing PD delivery, they may not consistently target key biological determinants of RKF preservation, particularly cumulative glucose exposure. Accordingly, minimizing cumulative glucose exposure rather than merely reducing exchange number may represent a more biologically relevant approach to defining and implementing IPD.

Consistent with the updated ISPD recommendations for PD prescribing, IPD should be implemented with careful monitoring of RKF and timely adjustment of dialysis dose to avoid underdialysis [2]. To enhance clinical applicability, a structured framework for IPD is summarized in Table 3. Overall, IPD may represent a physiologically plausible and patient-centered approach to dialysis prescription, particularly when aligned with strategies to minimize cumulative glucose exposure. However, given the predominantly observational nature of the available data, this concept should be interpreted with caution and requires further prospective validation.

**Table 3:** Clinically actionable framework for IPD.

Domain	Key components
Definition	IPD could be defined as a strategy that combines RKF with a reduced dialysis prescription to achieve solute and fluid removal targets while minimizing cumulative glucose exposure, with planned reassessment and timely step-up when targets are no longer met.
Patient selection	Preserved RKF; meaningful urine output; stable volume status; absence of uncontrolled hyperkalemia, metabolic acidosis, or uremic symptoms; adequate patient adherence and ability for close follow-up.
Monitoring	Reassessment of RKF, urine volume, volume status, and biochemical parameters every 1–3 months; earlier evaluation after peritonitis, hospitalization, abrupt changes in urine output, or clinical instability.
Step-up triggers	Progressive decline in RKF or urine output; failure to achieve adequate solute or fluid control; recurrent hyperkalemia or metabolic acidosis; development of uremic symptoms; increasing reliance on hypertonic glucose exchanges.
Key principle	Minimize cumulative glucose exposure while maintaining adequacy and clinical stability.

#### PD modality

APD offers flexibility and convenience, but its physiological characteristics raise important questions regarding RKF preservation. Evidence comparing APD and continuous ambulatory peritoneal dialysis (CAPD) regarding RKF preservation is inconsistent in the literature. In 2011, the NECOSAD study group reported that the risk of losing RKF is higher for patients starting dialysis on APD than for those starting on CAPD, especially in the first year [23]. Similarly, starting PD with APD has been associated with a faster decline of RKF and a higher risk of developing anuria than doing so on CAPD in an observational multicenter study [24]. On the other hand, the *bal*ANZ study group showed that PD modalities were not associated with RKF loss or urine volume in 2017 [25]. Despite these ongoing controversies, two meta-analyses published in 2007 and 2024 that evaluated randomized controlled trials found no clear difference between APD and CAPD with respect to preservation of RKF [26, 27].

However, these divergent findings likely reflect substantial heterogeneity across studies. Key sources of heterogeneity include differences in prescription intensity, UF strategies, cumulative glucose exposure, patient selection, and baseline RKF, as well as variability in definitions and measurements of RKF and in the duration of follow-up. Based on the currently available clinical evidence, it is difficult to conclude that CAPD is superior to APD in preserving RKF.

More importantly, modality-based comparisons may be inherently limited, as APD and CAPD represent delivery platforms rather than biologically distinct interventions. A more clinically relevant perspective may be to focus on modifiable treatment-related factors, such as cumulative glucose exposure, volume management strategies, and avoidance of hemodynamic instability. Accordingly, strategies aimed at protecting RKF may depend less on dialysis modality itself and more on individualized treatment approaches targeting modifiable factors, although this remains a conceptual perspective rather than a conclusion supported by definitive evidence.

#### Biocompatible solutions and icodextrin

The use of biocompatible PD solutions has been proposed as a strategy to mitigate systemic and intraperitoneal inflammation, thereby contributing to the preservation of RKF. Compared with conventional glucose-based solutions, biocompatible fluids are characterized by neutral pH, reduced concentrations of GDPs, and lower buffering-related cytotoxicity, which collectively may attenuate oxidative stress and endothelial dysfunction [28]. The *bal*ANZ study showed that biocompatible solutions resulted in 27% better RKF preservation [25]. The other randomized controlled study confirmed that biocompatible PD solution was associated with slower rates of decline in RKF [29]. In 2018, a Cochrane review found that biocompatible solutions improve RKF and urine volume preservation with high certainty [19]. However, these findings should be interpreted with caution, as the beneficial effects of biocompatible solutions are likely mediated through reduced exposure to GDPs, attenuation of intraperitoneal and systemic inflammation, and improved hemodynamic stability, rather than a direct intrinsic effect on RKF.

Icodextrin is a glucose polymer, that primarily provides improved UF through lower glucose exposure. By providing sustained osmotic pressure during long dwell exchanges, icodextrin may enable effective fluid removal without the need for high glucose concentrations, thereby reducing cumulative glucose load and its associated metabolic and inflammatory consequences. A preliminary study published in 2024 reported that icodextrin-based PD solutions were associated with a significant reduction in oxidative stress markers, along with a nonsignificant decrease in interleukin-6 levels [30], although the clinical relevance of these findings remains to be established.

On the other hand, some studies have suggested that increased UF capacity may be associated with adverse effects on RKF. The *bal*ANZ study showed that each 1 l/day increase in UF was associated with 8% worse RKF preservation [25]. But icodextrin usage was not specified in this study, and this association may reflect higher cumulative glucose exposure or more aggressive volume management strategies rather than UF per se. In a randomized controlled trial, Chang *et al*. reported that icodextrin was associated with better preservation of RKF compared with glucose-based solutions [31]. In contrast, a 2014 Cochrane review found no significant effect of icodextrin on RKF, while an updated 2018 review reported improved UF and reduced volume overload without adverse effects on RKF [19, 32].

Taken together, these findings suggest that the effects of biocompatible solutions and icodextrin on RKF are likely context-dependent. Some of the observed benefits may be mediated indirectly through improved volume control, reduced need for hypertonic glucose exchanges, or lower exposure to GDPs, rather than direct nephroprotective effects. From a clinical perspective, these strategies may help preserve RKF when used to minimize cumulative glucose exposure; however, their application should be individualized. In particular, excessive UF or overly aggressive volume management may predispose to intravascular volume depletion and renal hypoperfusion, potentially offsetting their benefits. Therefore, careful adjustment of UF targets, close monitoring of volume status, and integration with overall PD prescription are essential when using these approaches.

#### Blood pressure and volume management

Adequate blood pressure control plays a central role in the preservation of RKF in patients undergoing PD. Both sustained hypertension and excessive blood pressure reduction may adversely affect renal perfusion, emphasizing the need for balanced and individualized management strategies [33]. In PD, blood pressure control is closely linked to volume status, sodium balance, and UF efficiency, rather than dialysis modality alone. Accordingly, gradual volume control, avoidance of unnecessary dialysis intensification, and optimization of antihypertensive therapy are key principles to minimize intravascular depletion and renal ischemia.

Sodium removal is also critical, as interstitial sodium accumulation contributes to extracellular volume expansion and salt-sensitive hypertension, which may further accelerate RKF decline [34, 35]. Therefore, strategies proposed to improve blood pressure control in patients undergoing PD include careful dry-weight assessment, dietary sodium restriction, adjunctive use of loop diuretics, rearrangement of the PD regimen to individual peritoneal transport characteristics, and the use of icodextrin-based solutions; collectively, these approaches may also contribute to the preservation of RKF and peritoneal membrane integrity [34–37].

Diuretics are frequently used in PD patients with RKF to enhance urinary sodium and water excretion, thereby improving volume control and potentially reducing reliance on hypertonic glucose-based UF. However, their impact on RKF remains uncertain, with prior studies yielding inconsistent findings. Several studies reported that diuretic therapy led to higher urine output with no effect on GFR or creatinine clearance [38, 39]. Conversely, two studies conducted in Taiwan observed a more rapid decline in RKF associated with diuretic use [14, 40]. These conflicting findings likely reflect heterogeneity in patient selection, diuretic dosing strategies, baseline volume status, and concomitant use of renin–angiotensin–aldosterone system (RAAS) inhibitors. Importantly, increased urine output should not be interpreted as preserved RKF, as solute clearance and nephron integrity may continue to decline. Accordingly, whether the increase in urine volume achieved with diuretics translates into clinically meaningful benefits for PD patients remains uncertain.

Given that a substantial proportion of PD patients remain subclinically hypervolemic, with reported prevalence ranging from 47% to 67%, the judicious use of diuretics in selected patients may improve blood pressure control and indirectly support RKF preservation by reducing the need for hypertonic glucose-based exchanges [37, 41].

#### RAAS blockade

Blockade of the RAAS should be regarded as a cornerstone of blood pressure management in patients undergoing PD, and as a key strategy for the preservation of RKF. RAAS inhibitors, including angiotensin-converting enzyme inhibitors (ACEi) and angiotensin receptor blockers (ARBs), exert renoprotective effects beyond blood pressure lowering by reducing intraglomerular hypertension, attenuating proteinuria, and limiting progressive glomerulosclerosis. Several observational studies and randomized trials in PD populations have demonstrated a slower rate of RKF decline and prolonged urine output in patients treated with RAAS blockade compared with other antihypertensive agents [6].

A meta-analysis of six randomized controlled trial demonstrated that both ACEi and ARBs had additional and similar benefits in preserving RKF compared with other antihypertensive medications [42]. Another meta-analysis in 2017 showed that therapy with ACEi and ARBs is associated with a slower decline in RKF. Importantly, neither ACEi nor ARBs were associated with an increased risk of adverse events [43]. Consequently, unless contraindicated, RAAS inhibition should be considered an integral component of individualized blood pressure control strategies in PD patients, particularly in those with preserved RKF.

#### Sodium–glucose cotransporter-2 inhibitors

The clinical use of sodium–glucose cotransporter-2 (SGLT2) inhibitors in PD remains incompletely defined. The rationale for their use in PD includes potential hemodynamic stabilization, reduction in intraglomerular pressure, and modulation of inflammatory and metabolic pathways, which may theoretically support preservation of RKF [44, 45].

Available evidence in PD populations is limited and derived primarily from small observational studies and short-term analyses. These studies have generally reported neutral effects on UF capacity and peritoneal transport characteristics, without consistent evidence of clinically meaningful changes in RKF [46]. Some reports suggest potential benefits in volume control or metabolic parameters; however, these findings remain heterogeneous and should be interpreted with caution [46].

The use of SGLT2 inhibitors in PD should be considered exploratory, and their potential role in RKF preservation remains hypothesis-generating pending further clinical trial result.

#### Continuation of disease-modifying therapies in PD

An important clinical consideration in patients initiating PD is whether disease-modifying therapies introduced in earlier stages of chronic kidney disease should be continued after transition to dialysis, particularly in those with glomerular diseases. At present, evidence to guide continuation of such therapies in PD populations remains limited [2, 47, 48]. While ongoing treatment may offer potential benefits through modulation of disease-specific pathways and systemic effects, these must be balanced against uncertain efficacy in advanced kidney failure, as well as potential safety concerns in the dialysis setting [49].

In this context, continuation of therapy should be guided by a careful risk–benefit assessment. The potential advantages of ongoing treatment must be weighed against the risk of adverse effects, which may be amplified in patients receiving dialysis. Particular attention should be paid to the safety profile of each agent, including infection risk, metabolic complications, and drug-specific toxicities. In addition, altered pharmacokinetics and pharmacodynamic responses in PD patients should be considered, as these may influence both efficacy and tolerability. Accordingly, decisions regarding continuation of disease-modifying therapies should be individualized, taking into account the underlying diagnosis, prior disease trajectory, RKF, comorbidities, and overall treatment goals. Close monitoring and periodic reassessment are essential in this setting. Further studies are needed to better define the role and safety of these therapies in patients receiving PD.

#### Prevention of peritonitis

Given the evidence, reducing the frequency of peritonitis is crucial for preserving RKF in PD patients. Strategies such as proper aseptic technique, regular patient re-training, and prophylactic antibiotic use play a key role in preventing infections and thereby protecting RKF in accordance with the ISPD guideline [50]. These interventions not only reduce infection rates but also help maintain hemodynamic stability and limit unnecessary glucose exposure.

Aminoglycosides are frequently used to treat PD-associated peritonitis and bloodstream infections due to their broad gram-negative coverage and synergistic potential when combined with cephalosporins. The ISPD recommends aminoglycosides as part of empirical therapy for suspected PD peritonitis [50]. Studies evaluating the impact of aminoglycosides on RKF have not demonstrated a significant effect on the rate of RKF decline [51–53]. Despite their efficacy, aminoglycosides carry a risk of ototoxicity, particularly with prolonged or repeated use [54]. Oral N-acetylcysteine has been suggested by ISPD guidelines to mitigate ototoxicity, although it does not prevent RKF loss [50]. Overall, aminoglycosides remain a valuable treatment option, and early recognition and timely antimicrobial therapy are essential to prevent morbidity and preserve RKF.

#### Post-transplant management

Available evidence suggests that PD is a feasible option in this population, with comparable outcomes to other dialysis modalities [55–58]. In patients initiating PD following kidney allograft failure, management of immunosuppressive therapy represents an important clinical consideration; however, available evidence is limited and largely based on observational data and expert opinion.

Continuation or gradual tapering of immunosuppressive agents after allograft loss may attenuate inflammatory activation and reduce the risk of graft intolerance syndrome, a condition often associated with systemic inflammation and accelerated loss of RKF [59]. However, evidence guiding the optimal approach to immunosuppressive withdrawal remains heterogeneous and of low certainty, and is derived primarily from retrospective studies and clinical experience.

In cases of early graft failure (e.g. within the first year after transplantation), allograft nephrectomy and rapid withdrawal of immunosuppressive therapy may be considered in selected patients, particularly when the risks of infection or malignancy outweigh potential benefits [61]. In this setting, preservation of RKF may be less feasible. In contrast, in patients with later graft failure, continuation of low-dose immunosuppression for a limited period may be considered to mitigate inflammatory complications and potentially support RKF. For example, some centers use reduced-dose calcineurin inhibitors (e.g. tacrolimus ~2–5 ng/ml), although such targets should be interpreted as illustrative rather than prescriptive. Similarly, corticosteroids may be tapered over several months depending on clinical context [62]. During this period, RKF, urine output, and clinical status should be closely monitored. Withdrawal of immunosuppressive therapy may be considered in the setting of anuria or absence of graft intolerance symptoms, while antimetabolite agents are often discontinued earlier after dialysis initiation [60]. It is important to note that prolonged immunosuppression may increase susceptibility to peritonitis and contribute to peritoneal membrane injury, thereby indirectly compromising RKF preservation.

Overall, management of immunosuppression after allograft failure should be individualized, balancing potential benefits in preserving RKF and preventing inflammatory complications against the risks of infection, malignancy, and metabolic adverse effects. Given the predominantly observational nature of the available evidence, these strategies should be interpreted with caution, and further prospective studies are required to better define optimal management in this setting.

#### Treatment adherence

Treatment nonadherence is a common and clinically relevant issue in PD, with observational studies reporting varying degrees of nonadherence to dialysis exchange (2.6%–53%), medications (3.9%–85%), aseptic technique (50%), and dietary and fluid restrictions (14.4%–67%) [63, 64]. Inadequate adherence may contribute to suboptimal volume and sodium control, increased risk of peritonitis, and greater reliance on intensified dialysis prescriptions, all of which can adversely affect RKF. Although a direct causal relationship between nonadherence and loss of RKF has not been conclusively established, available evidence suggests that improving treatment adherence through patient education, supportive monitoring, and individualized PD regimens may play an important role in preserving RKF [65].

### Implementation and monitoring

#### Measurement of RKF

RKF in patients undergoing PD can be assessed using several complementary approaches, including urine volume, renal solute clearance, and measured GFR. Each method captures different physiological aspects of kidney function and may influence clinical interpretation.

Urine volume is the simplest and most widely used parameter in clinical practice, with thresholds such as >100 ml/day often considered clinically meaningful [5]. However, urine volume alone does not reflect solute clearance capacity and may overestimate preserved kidney function. Renal urea and creatinine clearance, typically obtained from timed urine collections, provide a more quantitative assessment and are commonly incorporated into total dialysis adequacy calculations [6]. Averaging urea and creatinine clearances provides a pragmatic estimate of residual clearance, though this approach remains influenced by collection accuracy and patient adherence.

Measured GFR using exogenous filtration markers represents the most accurate method, but is rarely feasible in routine clinical practice. As a result, most centers rely on serial measurements of urine volume and renal clearances to monitor RKF trajectories over time. Importantly, variability in RKF definitions across studies complicates the comparison of outcomes and the interpretation of evidence. Therefore, consistent use of standardized measurement approaches within clinical practice and research settings is essential to enable meaningful longitudinal assessment and improve comparability across studies.

#### Monitoring strategies

Preservation of RKF requires longitudinal monitoring strategies that extend beyond static or intermittent assessment. In routine clinical practice, urine volume and renal clearance should be reassessed at regular intervals, typically every 1–3 months, depending on baseline RKF and clinical stability. More frequent evaluation may be warranted following episodes of peritonitis, hospitalization, or significant changes in dialysis prescription.

In addition to direct RKF measurements, surrogate clinical indicators should be integrated into monitoring frameworks. These include trends in blood pressure, body weight, UF volumes, and symptoms suggestive of volume depletion or overload. Sudden changes in these parameters may signal an impending decline in RKF and prompt early intervention. A proactive monitoring approach should also incorporate systematic review of medication exposure, particularly nephrotoxic agents, and evaluation of cumulative glucose burden associated with PD prescriptions. Adjustments in dialysis intensity, fluid management, and pharmacological therapy should be guided by longitudinal trends rather than isolated measurements.

Ultimately, effective RKF monitoring requires a shift from episodic assessment to continuous, data-informed clinical decision-making, enabling early detection of risk patterns and timely individualized interventions.

#### Role of RM and AI

RM technologies and digital health platforms have emerged as promising tools in PD care, with evidence demonstrating benefits in reducing hospitalization, improving treatment adherence, and enabling earlier detection of clinical deterioration [66–69]. However, direct evidence linking RM to preservation of RKF remains limited, and current data should be interpreted primarily in terms of indirect or surrogate outcomes rather than definitive RKF benefit.

RM modules in PD typically involve connected cyclers, cloud-based data transmission, and clinician dashboards that provide real-time or near-real-time access to treatment parameters such as UF volumes, dwell times, alarm events, blood pressure, body weight, and treatment adherence [68]. These capabilities may facilitate earlier identification of volume imbalance, underdialysis, and treatment deviations, allowing timely adjustments in PD prescription and supportive therapies. In this way, RM may indirectly support RKF preservation by helping clinicians avoid hypovolemia, hypotension, and other acute stressors that accelerate RKF decline.

Beyond conventional RM, AI and machine-learning (ML) approaches may further enhance this framework by integrating longitudinal clinical and treatment data to identify patients at risk of accelerated RKF decline. Traditional statistical models often fail to capture the dynamic interactions between clinical variables, dialysis parameters, comorbid conditions, and patient behavior. ML algorithms such as random forests, gradient boosting machines, and neural networks can integrate large, longitudinal datasets generated by RM systems to identify hidden patterns and predict individualized risk of RKF loss, hospitalization, or technique failure.

Preliminary studies have demonstrated the feasibility of ML-driven prediction models using routinely collected PD data, including UF profiles, blood pressure trends, biochemical parameters, and peritonitis episodes [70–72]. However, these applications remain largely investigational, and their impact on RKF preservation has not yet been established in prospective studies. Accordingly, their role should be considered hypothesis-generating rather than evidence-based at present.

In this context, RM/AI may be better conceptualized as an enabling platform that supports implementation of established RKF-preserving strategies rather than a standalone intervention. A pragmatic “RKF-preservation bundle” may include optimizing of volume status, avoiding hypovolemia, minimizing cumulative glucose exposure, preventing peritonitis, and reducing nephrotoxic exposures. RM systems may facilitate this approach by enabling continuous tracking of relevant clinical and treatment parameters and supporting earlier, data-informed interventions (Table 4).

**Table 4:** Minimal RKF-preservation bundle and its clinical operationalization in PD.

Component	Clinical target	Practical approach	Potential supportive role of RM/AI*
Volume management	Avoid hypovolemia and large fluid shifts	Individualize UF targets; optimize diuretic use; adjust dietary sodium and fluid intake; prefer icodextrin for UF instead of hypertonic glucose solutions	Monitoring of body weight, blood pressure, and UF trends; early detection of volume imbalance
Glucose exposure	Minimize cumulative glucose burden	Consider IPD in appropriate patients; limit hypertonic exchanges; adjust prescription intensity	Tracking of dialysate glucose exposure and treatment patterns; identification of high-exposure profiles
Biocompatible solutions	Reduce glucose-related and GDP-mediated toxicity	Use low-GDP, neutral-pH solutions	Treatment-pattern tracking; identification of persistent bioincompatible exposure
Peritonitis prevention	Reduce infection episodes	Patient training and retraining; strict aseptic technique; early recognition and prompt treatment	Alerts for treatment deviations; adherence monitoring; early identification of clinical deterioration
Nephrotoxic drug avoidance	Prevent acute kidney injury and RKF loss	Regular medication review; avoidance of nephrotoxic agents (e.g. NSAIDs, contrast media) when possible	Medication tracking and clinical alerts
RKF monitoring	Detect decline early and guide therapy adjustment	Serial assessment of urine volume and/or renal clearance; integrate trends into prescription decisions	Longitudinal data integration; trend analysis; potential risk prediction using AI/ML models
Immunosuppressive therapy (post-transplant failure)	Modulate inflammation and prevent graft intolerance	Individualized tapering or continuation of low-dose IST; closely monitor for infection and adverse effects	Support for longitudinal monitoring and individualized treatment decisions
Primary disease management	Reduce disease-specific drivers of kidney injury	Optimize management of underlying disease (e.g. diabetes, autoimmune disease); minimize disease activity	Support for longitudinal data integration and identification of high-risk patterns

NSAIDs: nonsteroidal anti-inflammatory drugs; IST: immunosuppressive therapy.

*RM/AI are presented as supportive or enabling tools that may facilitate implementation of RKF-preserving strategies. However, direct evidence demonstrating their impact on RKF outcomes remains limited, and these approaches should be considered hypothesis-generating.

### Guideline perspectives and evidence gaps

Current international guidelines emphasize the importance of preserving RKF in patients undergoing PD, yet they largely address risk factors in isolation rather than as part of an integrated framework. Recommendations from the ISPD and the Kidney Disease Outcomes Quality Initiative focus on domains such as volume management, avoidance of hypovolemia, prevention of peritonitis, and minimization of nephrotoxic exposure, but do not capture the dynamic and interconnected nature of RKF decline [2, 73]. In this context, RM- and AI-based approaches should be conceptualized not as adjunctive technologies, but as unifying infrastructures that operationalize guideline principles across multiple domains of care.

Cumulative glucose exposure, a key but underrecognized determinant of RKF loss, should be considered a central modifiable mediator within this framework. While guidelines recommend limiting unnecessary glucose load and favoring more biocompatible solutions, they do not define cumulative glucose exposure targets nor provide mechanisms for longitudinal monitoring. RM-enabled PD platforms allow continuous capture of dwell prescriptions, UF efficiency, and solution use, generating granular datasets that reflect real-world glucose burden. AI-driven analytics can integrate these data with metabolic and inflammatory markers to identify patients at risk of glucose-mediated RKF decline, thereby translating general guideline principles into individualized, actionable strategies.

Similarly, volume control, widely acknowledged as a cornerstone of RKF preservation, remains largely guided by intermittent clinical assessments and static thresholds. Guidelines emphasize avoidance of hypovolemia but offer limited guidance on detecting subclinical dehydration or blood pressure variability over time. RM systems enable continuous surveillance of UF, body weight, and blood pressure, while AI-based models may help detect early hemodynamic instability and support proactive intervention. In this integrated model, digital tools function can be made as the operational extension of guideline-recommended volume management rather than as independent interventions.

Guideline recommendations are also limited in addressing patients returning to dialysis after allograft failure, a period characterized by clinical instability and rapid changes in RKF. In this setting, RM and AI may facilitate closer monitoring and individualized adjustment of dialysis and medical therapy.

Taken together, these observations highlight a central limitation of current guidelines: the lack of an integrated, data-driven framework linking modifiable determinants of RKF decline. RM and AI offer a potential infrastructure to bridge this gap by enabling continuous, patient-specific risk assessment. However, their role remains underrepresented in formal recommendations, largely due to the absence of prospective studies with RKF as a primary endpoint.

### Future directions

Future research in PD should move beyond isolated risk factor management toward integrated, RKF-centered strategies. A major evidence gap is the lack of prospective studies in which RKF preservation is defined as a primary endpoint rather than a secondary observation. Future randomized trials should compare RM-guided care with standard care using predefined RKF outcomes, while also incorporating event-driven endpoints to account for acute factors influencing RKF decline.

Mechanistic targets, such as cumulative glucose exposure, and volume status require more precise, longitudinal assessment. Glucose load, although widely recognized, is rarely quantified in clinical studies. Studies should develop methods to compute cumulative glucose exposure from cycler prescriptions and examine its association with RKF slope, independent of volume status and inflammation. Interventional research should test glucose-sparing strategies with explicit hypotheses about RKF preservation, rather than focusing solely on UF efficiency. Therefore, future research should prioritize the development and validation of standardized, mechanism-based definitions of IPD that move beyond simple metrics such as exchange number or fill volume, and instead focus on parameters directly linked to renal and peritoneal protection, including cumulative glucose exposure, solute clearance efficiency, and trajectories of RKF decline. Such an approach would facilitate more robust comparisons across studies, improve mechanistic insight, and support the design of clinical trials explicitly aimed at preserving RKF as a central therapeutic goal, ultimately informing evidence-based guidelines and optimizing patient-centered PD care.

Future research should evaluate whether RM-enabled, trend-based volume management can prevent subclinical hypovolemia and reduce RKF loss. This includes trials of individualized volume targets (dynamic rather than static), incorporating home blood pressure variability, weight trajectories, and UF patterns. Importantly, volume algorithms must be tested for unintended harms and should incorporate patient-reported outcomes as complementary signals.

The integration of AI into PD care requires a structured translational pathway that prioritizes validation, interpretability, and measurable clinical impact: external validation across centers, calibration to local practice, and prospective impact evaluation.

## CONCLUSION

Preserving RKF should remain a primary objective of PD care. Strategies such as IPD, individualized prescriptions, and minimization of cumulative glucose exposure may represent complementary approaches toward this goal, although supporting evidence remains heterogeneous and largely observational. Integrating physiological principles with emerging digital tools may offer a pathway toward more individualized and patient-centered PD care, but these approaches require further prospective validation.

## Data Availability

The data underlying this article are available in the article itself.
